# Complete Response to the Combination of Immunotherapy and Targeted Therapy in Stage IV Nasal Adenocarcinoma of a Dog: A Case Report

**DOI:** 10.1002/vms3.70861

**Published:** 2026-03-02

**Authors:** Yi Hu, Wanting Shi, Jingshu Xie, Shuaiyu Wang, Junli Feng, Youhong Su, Gebin Li

**Affiliations:** ^1^ China Agricultural University Veterinary Teaching Hospital Beijing China; ^2^ Small Animal Department College of Veterinary Medicine China Agricultural University Beijing China; ^3^ Biocytogen Pharmaceuticals (Beijing) Co., Ltd. Beijing China

**Keywords:** anti‐PD‐1 mAb, canine, combination treatment, TOC

## Abstract

Nasal adenocarcinoma is the most common nasal tumour in dogs, typically presenting with epistaxis, purulent discharge and sneezing. This malignancy often leads to local tissue invasion and, in advanced stages, neurologic symptoms. Without treatment, the prognosis of affected dogs is poor. While radiation therapy remains the standard treatment, its limited availability in some regions presents significant challenges. As a result, alternative treatments, including immunotherapy and targeted therapy, are gaining attention as viable options for improving outcomes in affected dogs. In March 2022, a 12‐year‐old uncastrated male West Highland white Terrier with a 1‐month history of chronic sneezing and unilateral epistaxis was referred for nasal tumour evaluation. Histopathologic examination revealed a nasal adenocarcinoma. Computed tomography indicated a 1.73 cm irregular soft tissue mass in bilateral nasal passages and frontal region with peripheral osteolysis but no local lymph node metastasis. The dog first received seven doses of anti–programmed death‐1 (PD‐1) monoclonal antibody (mAb) (MP‐001); after progression at 3 months (Day 91), we added seven doses of toceranib phosphate (Palladia) to the regimen. Remarkably, this treatment led to radiographic complete response (according to Veterinary Cooperative Oncology Group [VCOG] criteria), with the dog surviving 638 days from the initial diagnosis. This single case highlights the potential efficacy of combining anti‐PD‐1 mAb with Palladia in treating advanced nasal adenocarcinoma in dogs. Given the limited treatment options and poor prognosis for this aggressive cancer, this report suggests further investigation into such combination therapies may offer a promising alternative to conventional treatments like radiation in the future.

## Graphical abstract

The combination therapy of anti‐PD‐1 and TOC showed good therapeutic effects in this case of canine nasal adenocarcinoma, underscoring the potential of combining immunotherapy with targeted therapy in cases where single‐agent treatments fail. Combination therapy may enhance tumour growth inhibition by regulating the tumour immune microenvironment. It is imperative to conduct further research into the mechanisms and efficacy of this combined therapy approach in the future.

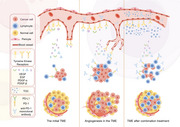

**Abbreviations**
CR: complete response
ICI: immune checkpoint inhibitor
mAb: monoclonal antibody
PD‐1: programmed death‐1
TKI: tyrosine kinase inhibitor
TME: tumour microenvironment
TOC: toceranib phosphate

## 1 Introduction

Nasal adenocarcinoma is the most common nasal tumour in dogs, predominantly affecting male dogs aged ≥ 10 years (MacEwen et al. 1977; Madewell et al. 1976; Lefebvre et al. 2005; Patnaik 1989). Dogs with nasal adenocarcinoma may show signs of epistaxis, purulent nasal discharge, sneezing and ocular discharges. As the disease progresses, it may locally invade the caudal region of the nasal cavity and cause neurologic signs. Regional lymph node (LN) metastasis rate is about 10%–24%, while pulmonary metastasis rate is about 2%–10% (Patnaik 1989; Henry et al. 1998; Rassnick et al. 2006; LaDue et al. 1999; Buchholz et al. 2009; Mason et al. 2013). Definitive diagnosis requires tissue biopsy and histopathology, with computed tomography (CT) commonly used to reliably assess the extent. Untreated Stage IV dogs have a median survival time (MST) of 3–7 months (Drees et al. 2009; Adams et al. 2009; Withrow et al. 2013).

Currently, radiation therapy (RT) is the treatment of choice, and chemotherapy, or a combination of both, are suboptimal options (Hahn et al. 1992; Langova et al. 2004; Woodruff et al. 2019). However, the limited availability of treatments for companion animals in China creates difficulty with standard treatment, leading to a need for alternative therapies. Therefore, immunotherapy, targeted therapy and combination treatment have become potential treatment methods for nasal adenocarcinoma (Maekawa et al. 2016; Ehling et al. 2022; Frezoulis and Harper 2022; Merino‐Gutierrez et al. 2021; Yamazaki et al. 2020).

Immune checkpoint inhibitors (ICIs) are a class of immunotherapy medications. ICIs, such as anti‐programmed death‐1 (PD‐1), have already had remarkable success in the treatment of various solid metastatic malignancies in both human and veterinary clinical settings (Maekawa et al. 2016; Stevenson et al. 2021; Shosu et al. 2016; Regan et al. 2016). Recent preclinical and clinical studies in human medicine have shown that antiangiogenic drugs, like tyrosine kinase inhibitors (TKIs), in combination with anti‐PD‐1/PD‐L1 therapy, can enhance the antitumour effect by altering the tumour microenvironment (TME), and combination therapy can significantly improve efficacy compared with single immunotherapy (Wallin et al. 2016; Zhao et al. 2019; Shigeta et al. 2020; Petrazzuolo et al. 2022).

Toceranib phosphate (TOC) (Palladia), a multitarget TKI, exerts antiangiogenic and antitumour effects by targeting multiple molecular targets (London et al. 2003; London et al. 2009). Additionally, TOC (Palladia) monotherapy has shown some efficacy in disease control of nasal adenocarcinoma (Merino‐Gutierrez et al. 2021; Chon et al. 2012).

These medications have potential as alternatives to radiation for nasal adenocarcinoma; however, their ultimate effectiveness is closely related to the TME, and no previous case has been reported on the efficacy of combined therapy.

Here, we report a case of Stage IV nasal adenocarcinoma that progressed after the initial treatment with canine anti‐PD‐1 monoclonal antibody (mAb) alone but achieved a complete response (CR) after receiving a combination treatment with TOC (Palladia) and anti‐PD‐1. This is the first reported case of using this combination in canine nasal adenocarcinoma, suggesting it may be an effective alternative to standard treatment for managing advanced cases.

## 2 Case Description

On March 27 2022, a 12‐year‐old, 9.4 kg uncastrated male West Highland White Terrier was presented with chronic sneezing and unilateral epistaxis. He was bright and alert with a good appetite on initial presentation. The only notable symptoms were frequent sneezing and nasal discharge. Besides a right nasal tenderness, the rest of the physical examinations were unremarkable. CT, surgical exploration and incisional biopsy were performed at a local clinic, revealing a space‐occupying lesion in the right nasal cavity and the left frontal sinus, which was initially suspected to be a nasal tumour.

The minimum database (complete blood count [CBC], serum biochemistry and urinalysis) was performed in our hospital. Only mild elevations of alkaline phosphatase (ALP) and proteinuria were noted. Prothrombin time and activated partial thromboplastin time (PT/APTT) were performed before invasive procedures and were within established reference ranges.

**FIGURE 1:**
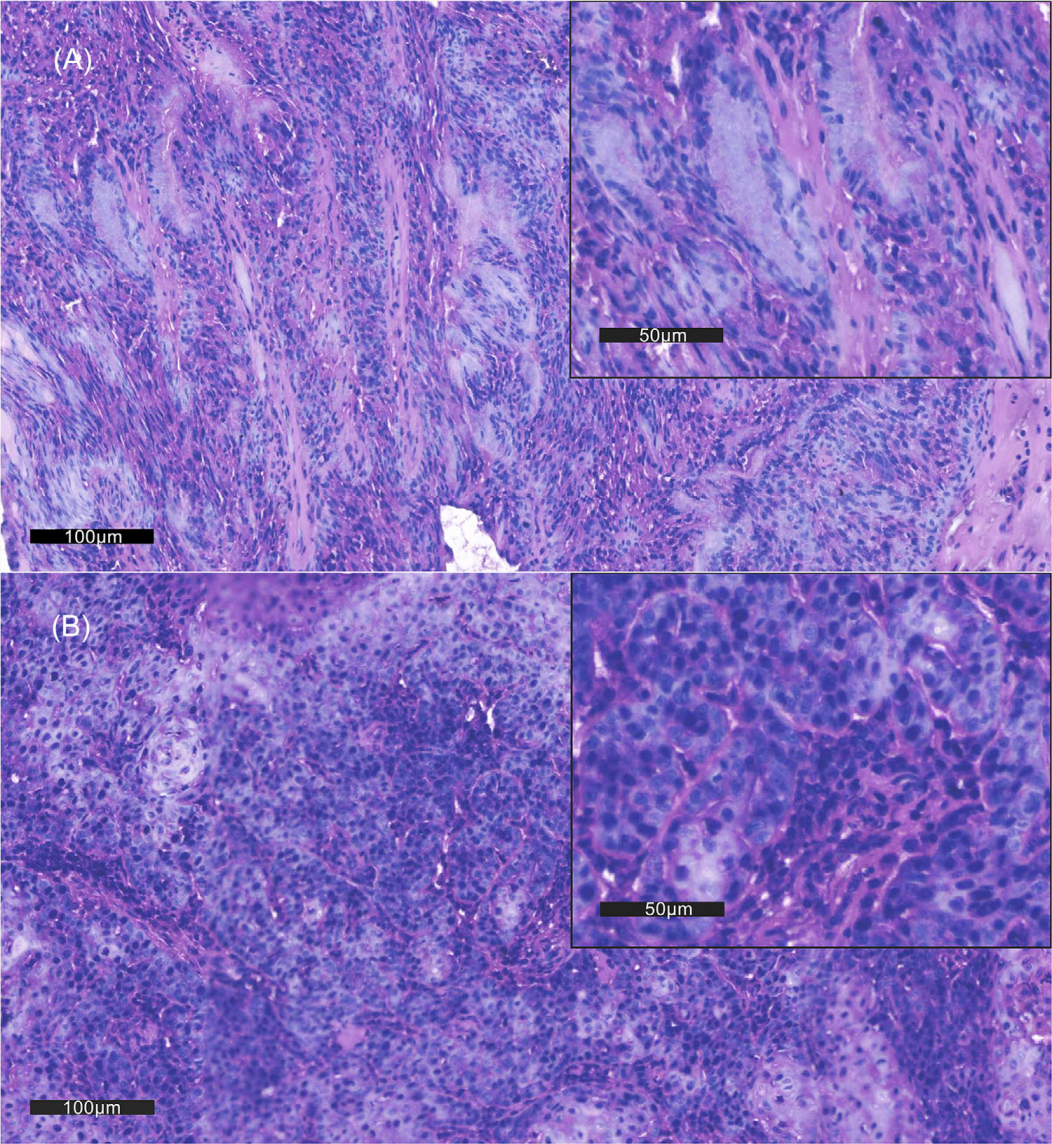
H.E. staining of tissue. (A) In the 20× magnification fields, these cells form dense sheets of acini and lumina containing basophilic secretions. Cells with squamous metaplasia are seen in some fields. (B) Local lymphocytic infiltration was observed. The number of lymphocytes in the stroma was greater compared with intratumoural sites. In the 40× magnification fields, tumour cells are polygonal, cuboidal and columnar, with indistinct cytoplasmic margins and abundant eosinophilic cytoplasm. Anisokaryosis, mitotic asymmetry and abnormal segregation of chromosomes were observed. There is medium nuclear pleomorphism, with coarse chromatin and mitoses. Severe infiltrates of neutrophils, lymphocytes and plasma cells are seen also.

**FIGURE 2:**
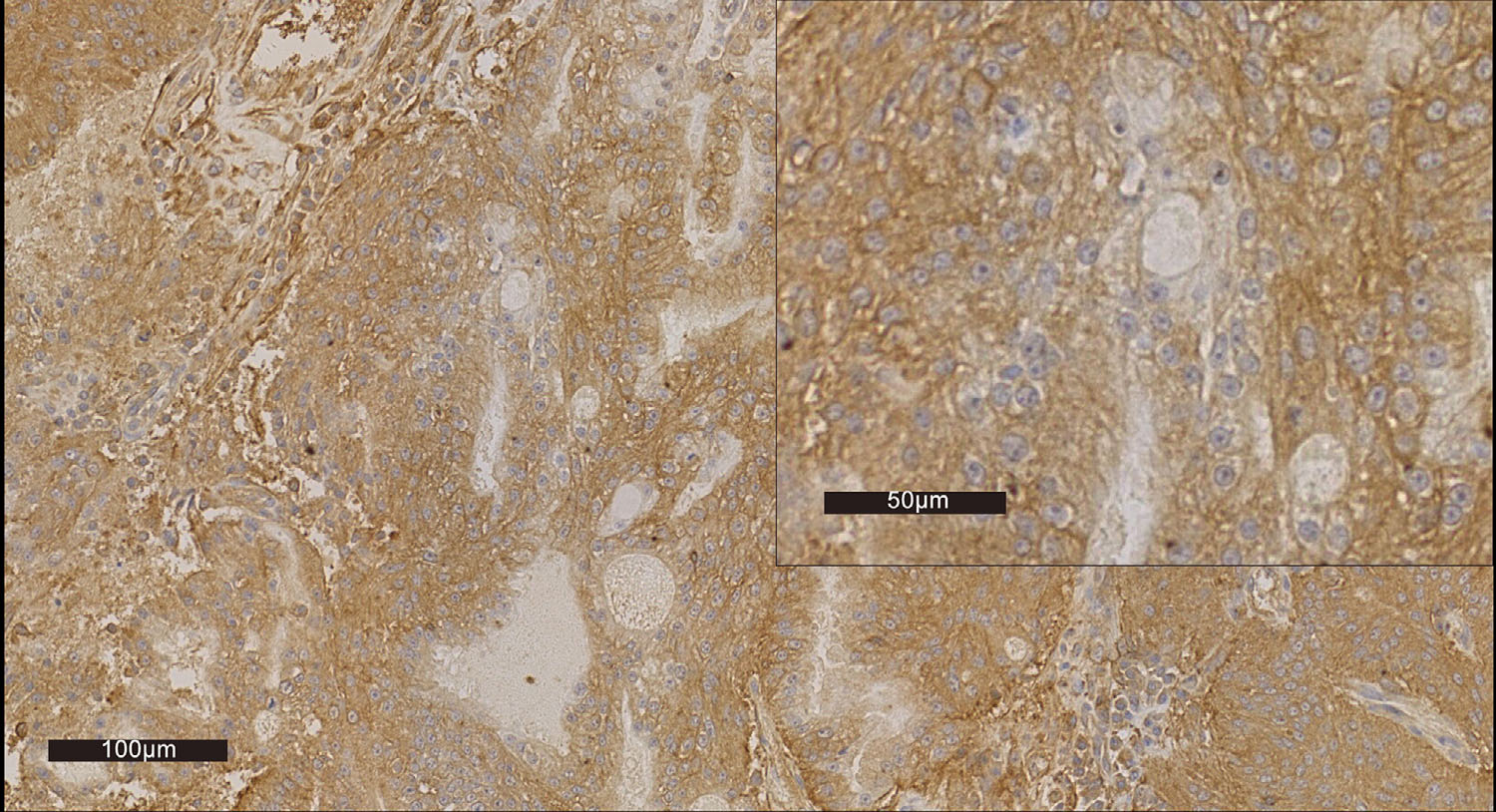
Immunohistochemical staining for PD‐L1 of tissue (anti‐PD‐L1 recombinant rabbit monoclonal antibody [JJ08‐95] [Huabio, Hangzhou, China], at a dilution of 1:100). Neoplastic cells formed the glandular appearance and were intensely positive on PD‐L1, especially for cell membranes (TPS ≥ 1%).

**FIGURE 3:**
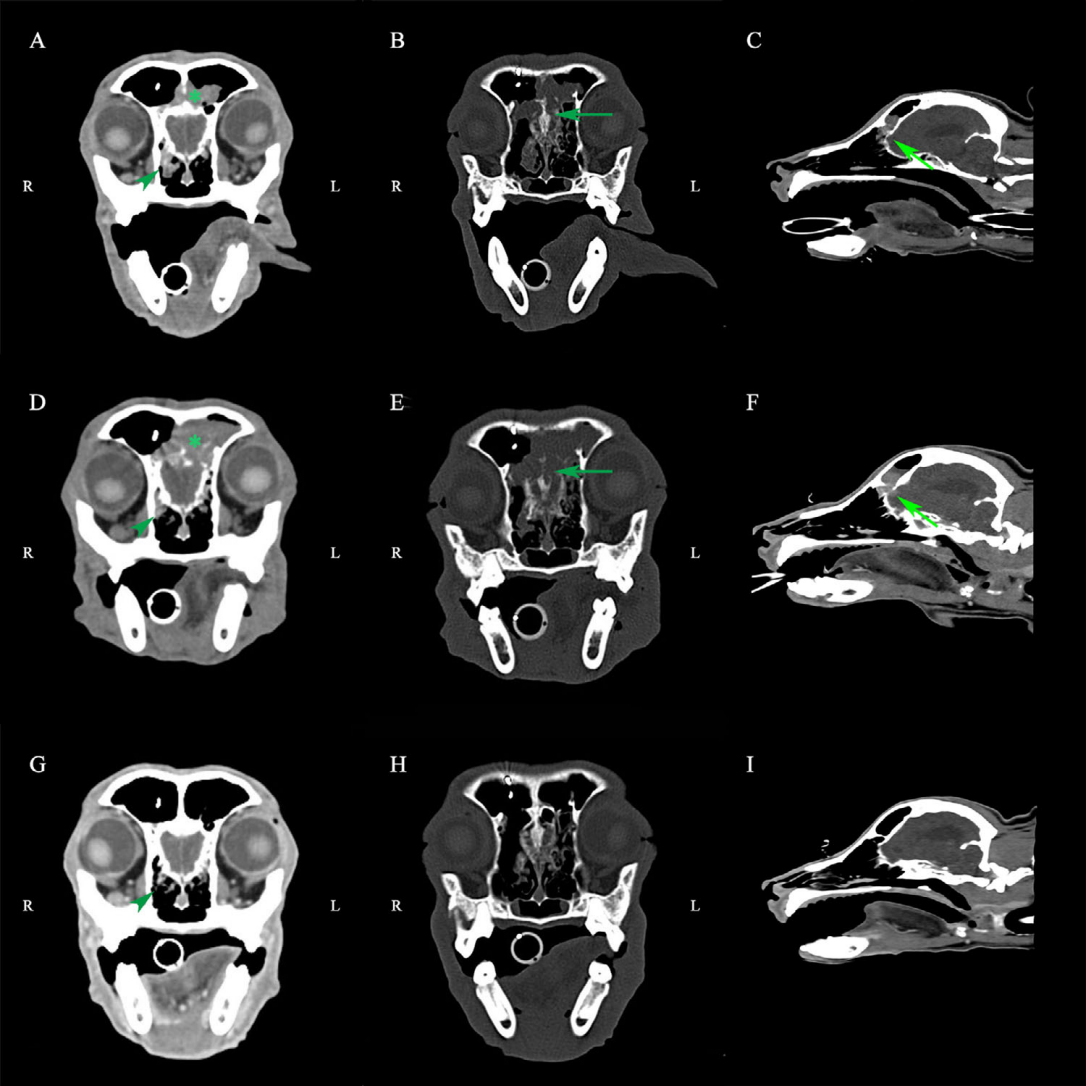
Nasal CT evaluation of the dog at baseline, after MP‐001 treatment alone and after MP‐001 combined with TOC (palladia) treatment. The arrowheads indicate the primary tumour. Arrows indicate ethmoid bone and cribriform plate invasion. (A–C) CT images at initial diagnosis. CT reveals a 1.73 cm nasal mass with bone erosion involving multiple sites, including the right mandible, turbinate and cribriform plate. (D–F) CT images after MP‐001 treatment alone for seven doses. CT confirming progression (53.8% size increase to 2.66 cm) showed extensive osteolysis of the bilateral ethmoid, right frontal/nasal and turbinate bones, with notable cribriform plate penetration and olfactory bulb invasion. (G–I) CT images after MP‐001 combined with palladia treatment for seven doses. Following treatment, CT confirmed a complete response (CR), with resolution of the frontal sinus and nasal cavity masses and improvement of the bone lesions in the ethmoid bone and cribriform plate.

We fixed frontal sinusotomy biopsy specimens in 10% neutral buffered formalin (24 h at 4°C), embedded them in paraffin, and processed them at our institutional histopathology lab. The neoplastic cells formed a glandular appearance with local lymphocytic infiltration, which confirmed a nasal adenocarcinoma (Figures 1 and 2). Further staging was conducted with abdominal ultrasound and CT. Gallbladder mucocele and bilateral renal cortical cysts with increased echogenicity were observed. There was no significant enlargement of the cervical or thoracic LNs, and no evidence of abdominal LN enlargement or organ invasion was observed. CT revealed a space‐occupying lesion in the right nasal cavity, the left dorsal nasal cavity and the bilateral frontal sinus. Tumour measurements were performed based on the sum of the longest diameters of target lesions, in accordance with cRECIST 1.1 guidelines (Nguyen et al. 2015). The maximum diameter of the target tumour was about 1.73 cm. The tumour was accompanied by surrounding bone lesions, involving the right jawbone, the right turbinate, the vertical plate of the bilateral palate and the cribriform plate, which was slightly dissolved (Figure 3A–C). Multiple local bone lyses were found in the right frontal bone, likely related to the biopsy procedure. Surgery‐related metal implant images were also observed. Multiple micronodules in the lung imaging were considered heterotopic ossification, and the remainder of the lung parenchyma was clear. Based on the clinical signs and results, a diagnosis of Stage IV nasal adenocarcinoma was made according to the modified Adams clinical staging method (Table 1) (Adams et al. 2009).

**TABLE 1:** Staging of nasal adenocarcinoma (Adams et al. 2009).

Stage	Characteristics
I	Confined to one nasal passage, paranasal sinus or frontal sinus, with no bone involvement beyond turbinates.
II	Any bone involvement (beyond turbinates), but with no evidence of orbit/subcutaneous/submucosal mass.
III	Orbit involved, nasopharyngeal, subcutaneous or submucosal mass.
IV	Tumour‐causing lysis of the cribriform plate.

Since RT is not widely available in China, three treatment options were offered to the owner: surgical excision followed by cytotoxic chemotherapy, targeted therapy and a clinical trial of anti‐PD‐1 treatment. The owner elected the anti‐PD‐1 trial.

The dog was treated with ‘MP‐001’, a canine anti‐PD‐1 mAb produced by Biocytogen Pharmaceuticals (Beijing) Co., Ltd., every 2 weeks at a dose of 3 mg/kg IV. The drug was diluted to 0.5 mg/mL with 0.9% NaCl and administered within 2 h. In addition, pamidronate disodium was given monthly to inhibit tumour‐induced osteolysis at a dose of 1 mg/kg IV. NSAID (Previcox, Boehringer Ingelheim Animal Health, Shanghai, China) was administered at a daily dose of 5 mg/kg PO for pain management. The liver and kidney indices were closely monitored throughout the treatment. Follow‐up abdominal ultrasounds were conducted every 2 months, and CTs were performed every 3 months to track the progression of the disease.

At 3 months, the dog progressed on MP‐001 monotherapy, with more frequent reverse sneezing and occasional epistaxis. CT showed a 53.8% increase in tumour diameter (1.73 → 2.66 cm) and extensive osteolysis with olfactory bulb invasion (cRECIST 1.0 by Veterinary Cooperative Oncology Group [VCOG]) (Nguyen et al. 2015). CT revealed extensive osteolysis involving the bilateral ethmoid bones, cribriform plate, right frontal and nasal bones, and turbinates, with evidence of olfactory bulb invasion and left frontal sinus effusion (Figure 3D–F). In response to these new findings, once we noticed the PD, we communicated with the client and adjusted the plan by adding TOC (Palladia, Pfizer Inc, Ascoli, Italy) into the treatment. MP‐001 and pamidronate disodium were given at the same frequency and dose, and TOC (Palladia) was given at 2.5–2.75 mg/kg PO EOD (Wallin et al. 2016; Zhao et al. 2019; Shigeta et al. 2020; Petrazzuolo et al. 2022; James and Dawn 2024). During the treatment, elevated urea and creatinine indexes were noticed. Gabapentin (Neurontin, Pfizer Inc, Ascoli, Italy) was substituted for the NSAID for pain control and was administered at 10 mg/kg PO BID due to concern of renal and gastrointestinal (GI) side effects of an NSAID in combination with TOC (Palladia) (Chon et al. 2012).

During the later period of treatment, the frequency of reverse sneezing gradually decreased, and no epistaxis was reported. After the seventh dose of the second serial treatment, CT showed that the space‐occupying lesions in the frontal sinus and nasal cavity had disappeared, and the image of bone invasion in the dorsal ethmoid bone and cribriform plate had been improved (Figure 3H–I). The dog achieved a CR (disappearance of all target lesions; pathologic LNs < 10 mm short axis) to the adjusted treatment (Nguyen et al. 2015).

**FIGURE 4:**
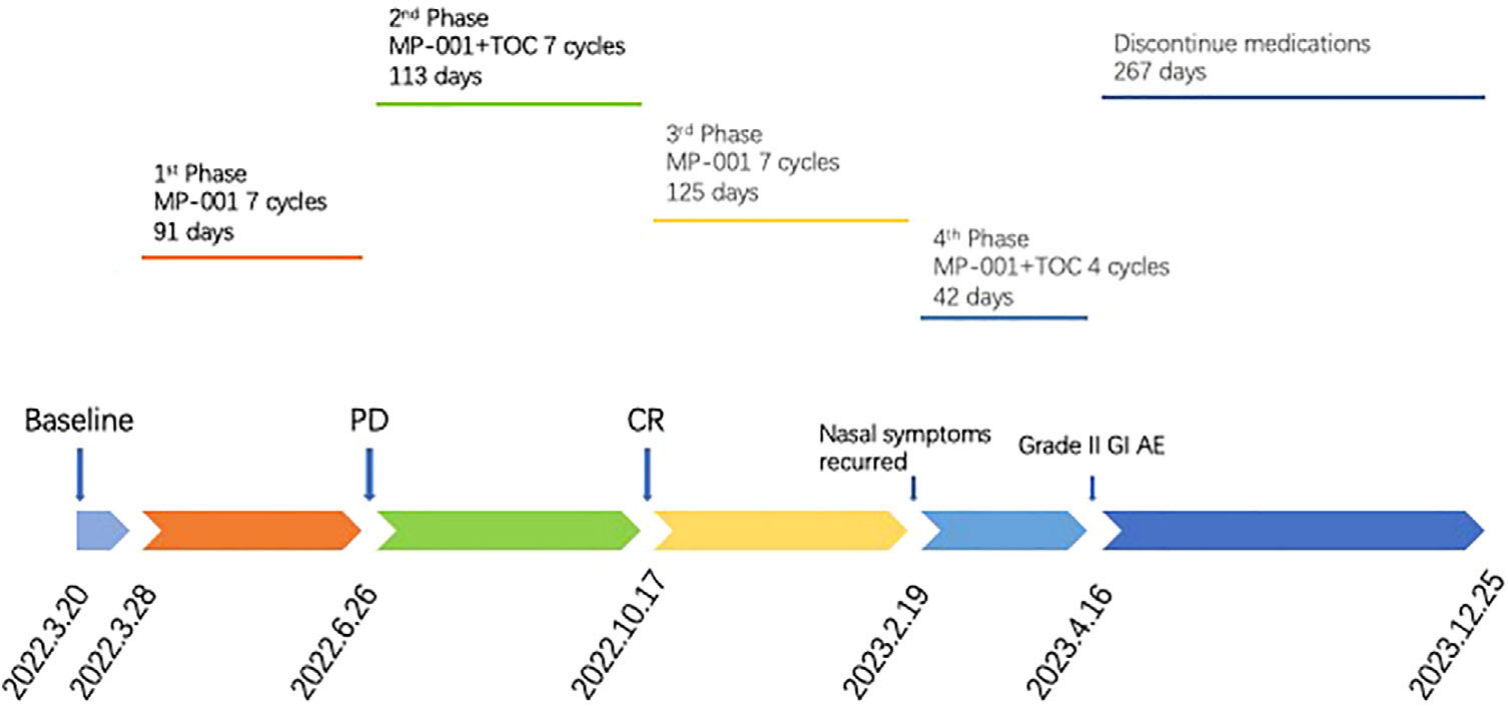
Timeline of treatment phases, medication changes and imaging outcomes.

However, the dog developed severe diarrhoea after adding TOC to the treatment. According to VCOG‐CTCAE v2, it was considered to be a Grade III GI adverse event (AE) of TOC (Table 2). Despite the treatment showing positive therapeutic effects, the GI symptoms persisted. Since the GI symptoms did not improve significantly after symptomatic treatment, we extended the TOC dosing interval from every other day to every third day. In cases of persistent signs, treatment was withheld until resolution before reintroducing the drug at the original dose (London et al. 2003; James and Dawn 2024; US Food and Drug Administration 2009). Once GI AEs did not improve significantly, TOC was discontinued in Month 7 (LeBlanc et al. 2021). Throughout the subsequent therapy, we had been balancing the antitumour efficacy with AEs of combination therapy. On April 16 2023, the client elected to discontinue all medications due to concerns about quality of life (QoL). There was no significant progression of nasal symptoms at follow‐up after all. However, the patient was euthanized later due to Stage IV chronic kidney disease on December 25 2023. The overall survival (OS) time is 638 days. Figure 4 provides a timeline of the key treatment phases, medication changes and imaging outcomes.

**TABLE 2:** Detailed case information.

Antitumour therapy	Dose	Treatment day (d)	Supportive therapy	Tumour diameter (response)	AE grade	Nasal symptoms	QOL impact	Treatment changes
—	0	7	—	1.73 cm (Baseline)	—	++	—	—
M	1	0	Pamidronate disodium NSAID (Previcox)	—	—	++	—	—
M	2	14	NSAID (Previcox)	—	—	++	—	—
M	3	28	Pamidronate disodium NSAID (Previcox)	—	—	++	—	—
M	4	42	NSAID (Previcox)	—	—	+++	—	—
M	5	64	Pamidronate disodium NSAID (Previcox)	—	Mildly elevated urea and creatinine indexes.	+++	—	Replace food with a nephropathy diet.
M	6	77	NSAID (Previcox)	—		+++	—	—
M	7	91	Pamidronate disodium NSAID (Previcox)	2.66 cm (PD)		+++	—	Add TOC (Palladia).
M + T	8	105	NSAID (Previcox)	—	Grade I GI AE mildly elevated urea and creatinine indexes.	++	—	—
M + T	9	120	Pamidronate disodium	—	Grade I GI AE mildly elevated urea indexes.	++	—	Discontinue NSAID (Previcox).
M + T	10	133	Gabapentin (Neurontin)	—	Mildly elevated urea and creatinine indexes.	+	—	Add gabapentin (Neurontin).
M + T	11	147	Pamidronate disodium gabapentin (Neurontin)	—	Grade III GI AE mildly elevated urea and creatinine indexes.	+	Vomiting, bloody stool and anorexia.	Discontinue TOC (Palladia) at the onset of bloody stools by the owner.
M + T	12	161	Gabapentin (Neurontin)	—	Mildly elevated urea and creatinine indexes.	++	Bloody stool but appetite restoration.	Discontinue TOC (Palladia) for 3 days due to bloody stools.
M + T	13	175	Pamidronate disodium gabapentin (Neurontin)	—	Mildly elevated urea, creatinine and phosphorus indexes.	+	Bloody stool.	Fluid therapy is recommended every 2–3 days. TOC (Palladia) was given every 3 days by the owner.
M + T	14	204	Gabapentin (Neurontin)	0 cm (CR)	Mildly elevated urea and creatinine indexes.	—	—	Discontinue gabapentin (Neurontin). TOC (Palladia) was withdrawn at the owner's request.
M	15	218	Pamidronate disodium	—		—	—	—
M	16	231		—		—	—	—
M	17	245	Pamidronate disodium	—		—	—	—
M	18	259		—		—	—	—
M	19	273	Pamidronate disodium	—		—	—	—
M	20	294		—		+	—	—
M	21	308	Pamidronate disodium	—	Moderately elevated urea and creatinine indexes.	+	—	—
M + T	22	329		—		++	—	Reuse TOC (Palladia).
M + T	23	343	Pamidronate disodium	—	Grade I GI AE moderately elevated urea and creatinine indexes.	++	A loss of appetite.	TOC (Palladia) was given every 3 days by the owner.
M + T	24	357		—		++	Vomiting, bloody stool and anorexia.	
M + T	25	371	Pamidronate disodium	—	Grade II GI AE moderately elevated urea and creatinine indexes.	+	—	TOC (Palladia) was discontinued by the owner.
M	26	385	—	—	Moderately elevated urea and creatinine indexes.	+	—	Discontinue all medications at the owner's request.
The case was euthanized later due to Stage IV chronic kidney disease with a survival time of 638 days.								

*Note*: M: MP‐001 only; M + T: MP‐001 + TOC.

## 3 Discussion

Advanced canine nasal adenocarcinoma carries a poor prognosis and poses significant treatment challenges. Currently, the recommended treatment for nasal and sinonasal tumours is RT. However, RT is not available in China for companion animals, forcing veterinarians to explore alternative therapies. Surgical excision by rhinotomy for local control has been associated with a high rate of recrudescence without significant lifespan prolongation, limiting surgery as the solitary treatment for nasal adenocarcinoma (MacEwen et al. 1977; Madewell et al. 1976; Henry et al. 1998). It was suggested that alternating carboplatin and doxorubicin combined with piroxicam therapy may be effective in the treatment of nasal adenocarcinoma but have a lower response rate than RT (Hahn et al. 1992; Langova et al. 2004; Woodruff et al. 2019). Therefore, postoperative adjuvant chemotherapy was proposed to be one of the alternative options superior to cytotoxic chemotherapy alone for treatment.

Targeted therapies, notably TOC (Palladia), have demonstrated antitumour and antiangiogenic activity in nasal adenocarcinoma (Gramer et al. 2017; Hocker et al. 2017). TOC (Palladia) is a TKI with both antitumour and antiangiogenic activity through the inhibition of vascular endothelial growth factor receptor (VEGFR), platelet‐derived growth factor receptor β(PDGFR‐β) and KIT, which are highly expressed in canine nasal adenocarcinoma (Gramer et al. 2017; Hocker et al. 2017). Considering the reported therapeutic effect of TOC (Palladia) in the treatment of nasal adenocarcinoma, target therapy was also initially given to the owner.

In addition, the TME of nasal adenocarcinoma is characterized by a high degree of lymphocyte infiltration and vascularization (Wang et al. 2018; Ferradini et al. 1991; Almangush et al. 2018). In this case, there was lymphocyte infiltration in the local area of the tumour (Figure 1). With the infiltration of lymphocytes, there is an upregulation of PD‐1 and PD‐L1 in the TME, which may suppress T cell receptor‐mediated cytotoxicity and CD8^+^T cell proliferation (Tang et al. 2022). To evaluate PD‐L1 expression, an anti‐PD‐L1 recombinant rabbit mAb (Huabio, Hangzhou, China) was utilized for staining at a dilution of 1:100. Stained sections were assessed using the tumour proportion score (TPS) system, with PD‐L1 positivity defined as ≥ 1% of tumour cells showing distinct membrane staining. The neoplastic cells formed a glandular appearance and were intensely positive on PD‐L1, particularly along the cell membranes (Figure 2), suggesting that the tumour was likely responsive to anti‐PD‐1 therapy. Anti‐PD‐1 treatment has shown to be effective in the treatment of canine malignant melanoma with limited AEs. Given the dog's Stage IV diagnosis and the poor prognosis associated with nasal adenocarcinoma, the use of immunotherapy was considered a reasonable starting point, especially in the absence of RT. MP‐001 is an anti‐PD‐1 mAb, which reduces immune suppression by blocking the binding of PD‐1 and PD‐L1. A clinical case has reported a partial response to MP‐001 treatment in dogs with salivary gland carcinoma (Xu et al. 2023). Therefore, MP‐001 was considered a viable treatment option.

To sum up, postoperative adjuvant chemotherapy, targeted therapy and immunotherapy were available as treatment options, and their advantages and disadvantages were listed in detail (Chon et al. 2012; Pan et al. 2016; Pellin et al. 2017; Wouda et al. 2018). Following discussion, the owner elected to move forward with immunotherapy.

However, during the first serial treatment, although immunohistochemical staining of tumour tissue for PD‐L1 was positive (Figure 2), the single usage of MP‐001 monotherapy did not achieve good efficacy. The failure of monotherapy highlighted the limitations of PD‐L1 expression as a standalone predictive biomarker (Shklovskaya and Rizos 2020). This is particularly evident in complex TMEs where other factors, such as angiogenesis, T‐cell infiltration and T‐cell proximity to tumour cells, tumour mutational load, presence of additional immune checkpoints and signatures of IFN‐γ response, can significantly influence the response to anti‐PD‐1 monotherapy (Toh et al. 2016; Ilie et al. 2017; Motz and Coukos 2013; Georganaki et al. 2018). These factors hold promise as biomarkers that complement PD‐L1 testing, meriting further research. In addition, the composition and density of tumour‐infiltrating lymphocytes (TILs) play a critical role in determining the efficacy of immunotherapy (Toh et al. 2016; Ilie et al. 2017). In the H.E. stained sections, lymphocytic infiltration was found primarily in the stroma, along with a large number of microvessels and ill‐formed vessels (Figure 1). Abnormal tumour vasculature impedes T‐cell extravasation and creates an immunosuppressive microenvironment, which TOC can normalize to enhance PD‐1 blockade ^4^8. In this case, we hypothesize that the addition of the targeted antiangiogenic agent (TOC) helped normalize tumour vasculature, remodel the TME and promote high endothelial venule formation, thereby facilitating T‐cell migration and enhancing tumour sensitivity to PD‐1 inhibition (Yamazaki et al. 2020; Wallin et al. 2016; Zhao et al. 2019; Shigeta et al. 2020; Ferradini et al. 1991; Pan et al. 2016; Pellin et al. 2017; Georganaki et al. 2018; Jain 2013; Rahma and Hodi 2019). This mechanistic rationale is supported by studies of other antiangiogenic agents (sunitinib, sorafenib, apatinib, bevacizumab, etc.) in human oncology, which have demonstrated improved outcomes when combined with anti‐PD‐1 antibodies across multiple malignancies (Petrazzuolo et al. 2022). The evidence stated above contributed to our second serial treatment. Given the dog's advanced stage, a combinatorial strategy targeting both immune evasion and angiogenic pathways was implemented. The marked clinical response following the introduction of TOC supports the therapeutic potential of dual PD‐1 and angiogenic inhibition. Nevertheless, the exact mechanisms through which TOC reprogrammes the TME remain speculative and warrant further investigation in dedicated preclinical and clinical studies. Performing re‐biopsies at different time points during treatment (while the tumour is still present) followed by histological and immunological examinations, including assessment of immune cell infiltration, vascular morphology, maturity and normalization, as well as exploration of immune cell re‐characterization, represents a feasible strategy for future implementation.

Following the introduction of TOC, the dog showed a remarkable response, achieving a CR of the tumour. This outcome is especially significant given the advanced stage of the disease, and it underscores the potential of combining immunotherapy with targeted therapy in cases where single‐agent treatments fail. In two retrospective reviews, it was reported that dogs with Stage IV nasal adenocarcinoma had an MST of 7 months. With signs of epistaxis, MST was approximately 3 months (Rassnick et al. 2006; Adams et al. 2009; Withrow et al. 2013). For our case, the prognosis would be poor to grave. With combination therapy, this patient survived 638 days after the initial diagnosis of nasal adenocarcinoma with CR of the target mass, which is significantly longer than the previously reported median OS (88 days) of dogs with nasal adenocarcinoma at Stage IV with epistaxis (Adams et al. 2009; Withrow et al. 2013). Based on previous research, the survival benefit of this case is better than the reported response to treatment with TOC alone, further reflecting the advantages of the combination therapy of anti‐PD‐1 mAb plus TOC (Merino‐Gutierrez et al. 2021; Chon et al. 2012; Bernabe et al. 2013; London et al. 2012). The MST of radiotherapy is usually between 12–18 months, which highlights the potential of this alternative therapy in areas where RT cannot be obtained (Hahn et al. 1992; Langova et al. 2004; Woodruff et al. 2019).

However, several important limitations of this study should be acknowledged. First, it is important to note that this case is only a single clinical finding of TOC plus anti‐PD‐1 therapy. To support this finding, more experiments in vitro and in vivo should be conducted to solidify the theory and provide more data. Second, it would be more convincing if the second biopsy sample could be collected before the combination therapy for analysis of the TME to provide a better rationale for combination therapy. Third, a CR was assessed radiographically according to VCOG criteria, but without final histopathologic confirmation. Although biopsy remains the gold standard for verifying CR, the decision was made in agreement with the owner to avoid invasive procedures, given the dog's excellent clinical condition and QoL. For similar future studies, cytologic or histologic confirmation should be actively sought when feasible.

Additionally, TOC tolerance exhibited significant interindividual variability (James and Dawn 2024). Although radiographic CR was achieved, Grade III GI AEs impacted daily function and warranted dose adjustments. This underscores the importance of balancing aggressive treatment with patient comfort. This led to the extension of the dosing interval and withdrawal of TOC during treatment, which may have an impact on therapy to a certain extent. On the contrary, despite discontinuing TOC, the dog's disease remained stable for several months, suggesting that the benefits of combination therapy can extend beyond the active treatment phase. However, it remains unclear what the residual therapeutic effect was attributable to. Based on established mechanisms of anti‐PD‐1 therapies, this sustained response is consistent with synergistic effects reported in the literature, whereby antiangiogenic agents promote TME remodelling and facilitate robust T‐cell activation and memory formation induced by immune checkpoint inhibition. Thus, the durability of benefit likely stems from this complementary action rather than either agent alone. Future studies should also be conducted on the relationship between treatment duration, AEs and the ongoing benefits of combination therapy. We recommend that future trials consider three treatment arms—anti‐PD‐1 monotherapy, TOC monotherapy and combination therapy, with the following endpoints: primary endpoints of objective response rate (ORR) and disease control rate (DCR), along with key secondary endpoints including CR rate, OS, progression‐free survival (PFS), safety profiles and QoL. This comprehensive set of endpoints would provide a robust assessment of both efficacy and tolerability, guiding optimal clinical development.

Besides, we performed an attribution analysis for the elevation in renal parameters in this case, including known toxicity profiles, temporal relationship analysis and treatment discontinuation challenge. We concluded that TOC and NSAIDs (probable causality) were the primary factors leading to nephrotoxicity. The former was implicated because its introduction immediately preceded the deterioration of parameters, while the necessity of the latter was demonstrated by the improvement in parameters after its discontinuation. Pamidronate (possible causality), as a drug with known nephrotoxicity, may have contributed background risk but was not the main cause. Conversely, for MP‐001 (unlikely causality), there is a lack of evidence for association. Therefore, following the onset of nephrotoxicity after TOC introduction, replacing the NSAID with gabapentin was a theoretically justified and clinically effective intervention. However, whether the combination of NSAIDs and TOC leads to an exacerbation of renal AEs remains debatable. Nonetheless, existing literature has suggested caution regarding the combined use of NSAIDs and TOC due to the potential for additive GI side effects (James and Dawn 2024; Zoetis Canada 2025). Combined with the potential renal side effects possibly indicated in this case, the safety of TOC plus NSAIDs concomitant use warrants prudent consideration. Indeed, future studies involving in vitro and in vivo experiments with monotherapy groups and pairwise drug combination studies are needed to investigate the side effects of combined drug use.

## 4 Conclusion

In summary, combined anti‐PD‐1 mAb and toceranib therapy induced a CR and 638‐day survival in Stage IV canine nasal adenocarcinoma. The notable and sustained response of the tumour to the combination of anti‐PD‐1 mAbs and TOC, in this case, may indicate potential value of this approach in nasal adenocarcinoma and possibly other malignancies. However, significant GI AEs were observed, underscoring the need to balance treatment efficacy with toxicity management. As these findings are derived from a single case, generalizability should be interpreted with caution. Prospective trials should assess long‐term outcomes, optimize dosing to balance efficacy and toxicity and incorporate standardized QoL metrics.

## Author Contributions

Y.H. contributed to the investigation, data curation, visualization and writing of the original and review drafts. W.S. assisted in the conceptualization and writing of the review draft. Y.H. and W.S. contributed equally to this work. S.W. participated in project administration and data curation. J.F. contributed to the investigation and data curation. J.X. and Y.S. focused on resource coordination and supervision. G.L. contributed to the conceptualization, project administration, resource coordination, supervision and writing of the drafts.

## Funding

The authors have nothing to report.

## Ethics Statement

The study was reviewed and approved by the Ethic Committee of China Agricultural University Laboratory Animal Welfare and Animal Experiment (issue no. Aw40103202‐2‐1). Written informed consent was obtained from the dog's owner for the publication of this case report.

## Conflicts of Interest

One of the authors, Jingshu Xie, was employed by the company Biocytogen Pharmaceuticals (Beijing) Co., Ltd. The remaining authors declare no conflicts of interest.

## Data Availability

The data that support the findings of this case are available from the corresponding author upon reasonable request.
